# A Deep Belief Network and Dempster-Shafer-Based Multiclassifier for the Pathology Stage of Prostate Cancer

**DOI:** 10.1155/2018/4651582

**Published:** 2018-03-19

**Authors:** Jae Kwon Kim, Mun Joo Choi, Jong Sik Lee, Jun Hyuk Hong, Choung-Soo Kim, Seong Il Seo, Chang Wook Jeong, Seok-Soo Byun, Kyo Chul Koo, Byung Ha Chung, Yong Hyun Park, Ji Youl Lee, In Young Choi

**Affiliations:** ^1^Department of Computer Science and Information Engineering, Inha University, InhaRo 100, Nam-gu, Incheon, Republic of Korea; ^2^Department of Medical Informatics, College of Medicine, The Catholic University of Seoul, 222 Banpo-daero, Seocho-gu, Seoul 06591, Republic of Korea; ^3^Department of Urology, University of Ulsan College of Medicine, Seoul, Republic of Korea; ^4^Department of Urology, Sungkyunkwan University School of Medicine, Seoul, Republic of Korea; ^5^Department of Urology, Seoul National University College of Medicine, Seoul, Republic of Korea; ^6^Department of Urology, Seoul National University Bundang Hospital, Seongnam, Republic of Korea; ^7^Department of Urology, Yonsei University College of Medicine, Seoul, Republic of Korea; ^8^Department of Urology, Seoul St. Mary's Hospital, College of Medicine, The Catholic University of Korea, Seoul, Republic of Korea

## Abstract

**Object:**

Pathologic prediction of prostate cancer can be made by predicting the patient's prostate metastasis prior to surgery based on biopsy information. Because biopsy variables associated with pathology have uncertainty regarding individual patient differences, a method for classification according to these variables is needed.

**Method:**

We propose a deep belief network and Dempster-Shafer- (DBN-DS-) based multiclassifier for the pathologic prediction of prostate cancer. The DBN-DS learns prostate-specific antigen (PSA), Gleason score, and clinical T stage variable information using three DBNs. Uncertainty regarding the predicted output was removed from the DBN and combined with information from DS to make a correct decision.

**Result:**

The new method was validated on pathology data from 6342 patients with prostate cancer. The pathology stages consisted of organ-confined disease (OCD; 3892 patients) and non-organ-confined disease (NOCD; 2453 patients). The results showed that the accuracy of the proposed DBN-DS was 81.27%, which is higher than the 64.14% of the Partin table.

**Conclusion:**

The proposed DBN-DS is more effective than other methods in predicting pathology stage. The performance is high because of the linear combination using the results of pathology-related features. The proposed method may be effective in decision support for prostate cancer treatment.

## 1. Introduction

Prostate cancer is the most common cancer in men, with around 1.1 million cases diagnosed and approximately 309,000 deaths in men worldwide in 2012 [1]. It is estimated that 40–50% of men may also have potentially extraprostatic disease [2].

Carcinectomy and radiotherapy are the typical treatments for prostate cancer [3]. The choice of treatment for prostate cancer requires extensive experience and analysis of treatment cases. Pathological staging is the process of predicting the likelihood of prostate cancer disease spreading in a patient prior to treatment. The clinical stage evaluation is based on data gathered from clinical tests that are available prior to treatment or the surgical removal of the tumor. Cancer staging evaluation occurs both before and after the tumor is removed: the clinical and pathological stages, respectively [4]. Pathologic staging is determined after the removal of the tumor tissue and after surgery. This is more likely to be more accurate than clinical staging because it evaluates the direct nature of the disease. Therefore, the prediction of pathological stages using clinical data analysis is an important factor in the treatment of prostate cancer [5].

Pathologic staging prediction is very important because it provides physicians with optimal treatment and management strategies. For example, radical prostatectomy (RP), the surgical removal of the prostate gland, provides the best opportunity for cure when prostate cancer is localized and accurate prediction of the pathology stage can provide the most beneficial treatment approach [6–8]. Currently, Partin tables are used to predict the prognostic clinical outcome for prostate cancer, which are based on statistical methods such as logistic regression [9, 10]. The Partin tables use clinical test data including prostate-specific antigen (PSA) level, Gleason score, and clinical T stage to predict the pathology stage. While the Partin tables have been verified from 2001 to 2011, there are questions about their applicability to current patients following environmental changes [11]. Thus, a new classification method using machine learning is needed to provide an accurate prediction of the pathology stage [12].

Deep belief networks (DBN) are a deep learning technique and is an effective method for classification prediction [13, 14]. As DBN supports both unsupervised and supervised learning, it is possible to effectively learn about uncertain data relationships [15, 16]. Because PSA level, Gleason score, and clinical T stage for stage prediction have uncertainties in each patient, a combination of evidence for each variable is needed. The Dempster-Shafer theory (DS) is a technique used to fuse information based on trust values [17, 18]. The DS allows the combination of evidence from different sources to arrive at a degree of belief (represented by a mathematical object called a “belief function”) that considered all available evidence [19, 20]. This technique is a method for fusing information using a stochastic calculation method for belief values [21]. This allows fusion of the classification results of each variable to the pathology stage.

In this paper, we propose a DBN-DS-based multiclassifier for pathologic stage prediction of prostate cancer. The proposed DBN-DS uses patient PSA level, Gleason score, and clinical T stage and three DBNs to predict the pathology stage by combining the predicted information from the classifier. The classifiers are created by learning data according to features. When output values are generated using each learned DBN classifier, the final predicted result is provided by stochastically calculating the predicted output from each DBN classifier using DS. This paper is organized as follows: Section 2 presents the proposed technique and its process.  Section 3 explains the experiments and presents their outcomes. Finally, Section 4 presents the conclusions.

## 2. Materials and Methods

### 2.1. Data Set

The study data comprised 6345 male patients extracted from the Korean Prostate Cancer Registry (KPCR) which is extended from Smart Prostate Cancer Data Base (SPCDB) at six tertiary medical centers in Korea [22]. The three input variables consist of initial PSA, Gleason score, TRUS volume, and clinical T stage. Two output variables consisting of pathologic T stage (pT2a, pT2b, pT3a, pT3b, and pT3c) and N stage (pN1) were used. The output variables are transformed using the guidelines of the American Joint Committee on Cancer (AJCC), which were used to identify the pathologic stage as organ-confined disease (OCD; pT2+) or non-organ-confined disease (NOCD; pT3+ or N+) [23]. For the experiments, the data from the KPCR were divided into a training set 70% (4039 patients) and a validation set 30% (2306 patients).

### 2.2. Deep Belief Network

A deep belief network (DBN) is a generative graphical model or a type of deep neural network composed of multiple layers of latent variables, with connections between the layers but not between the units within each layer. The DBN is composed of restricted Boltzmann machine (RBM) layers. The learning method in the DBN is done by configuring the visible layer and hidden layer 1 into a single RBM. The DBN is composed of multiple layers of RBMs [24]. The RBMs consist of visible and hidden unit layers. Once learning is complete, hidden layers 1 and 2 are trained via the RBM by giving a new input as a value of the hidden layer 1. As such, learning is performed up to the last layer sequentially [25]. One classification technique using the DBN is back propagation, which is configured in the uppermost layer in the DBN [26]. This technique shows better results than an artificial neural network (ANN), which uses a connection intensity that is arbitrarily selected.

**Figure 1:**
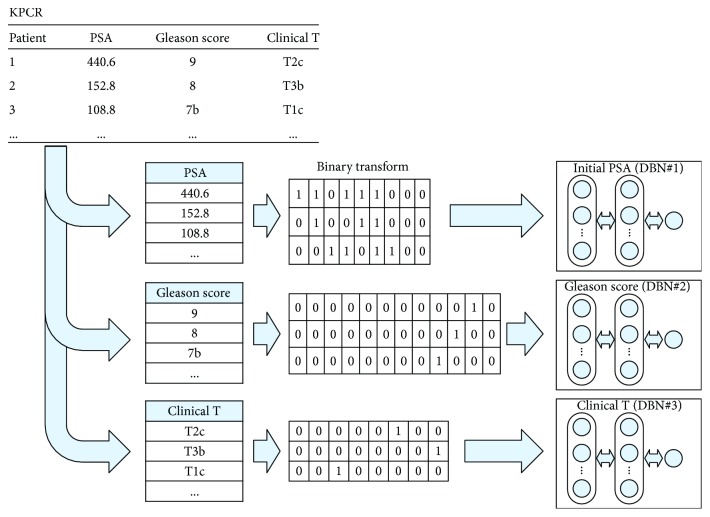
Multi DBN classifiers.

In this study, we constructed a classifier for three input and two output variables to construct a multiclassifier, as shown in Figure 1. We created one classifier for each variable. Our idea was to use multiclassifiers for each variable [27]. The purpose of this study was to make a linear combination of the predictions of the classifiers using DS [28]. Therefore, one variable must be converted into several input values. As PSA levels are continuous data, they were converted into binary numbers and configured as an input node. Because Gleason score and the clinical T stage are categorical data, they constitute an input node by constructing data in flag form.

### 2.3. Dempster-Shafer-Based Information Fusion

Dempster-Shafer (DS) is a mathematical theory that deals with the uncertainty and inaccuracy problems presented by Arthur Dempster and Glenn Shafer [29]. The DS provides an effective method for establishing evidence intervals using belief and likelihood values for the data set. The DS can support the combination of information. As a result, it is possible to use a combination rule to set various information as an evidence value and to calculate the result of all the evidence [30].

The DS expresses the degree of certainty as a section and sets mutually exclusive hypotheses such as probability. The set of objects is called the environment and is denoted by *θ*. The *θ* can have several elements such as *θ* = {*θ*1, *θ*2, *θ*3,…, *θk*}, and the number of subsets is 2^*k*^. When *θ* has only one element, it is called an identification frame. A set of 2^*k*^ subsets is called a power set and is denoted by *θ*. The degree to which *θ* is supported by any evidence is called the basic probability assignment function *m* (1). The *m* is mapped to a probability value of 0 for an empty set, and the sum of *m* is 1 for all subsets of *θ* (2).

(1) $$\begin{array}{c} m:2^{k}\to \left[0,1\right], \end{array}$$

(2) $$\begin{array}{c} m\left(\emptyset \right)=0, \end{array}$$

Belief (*H*), which is the belief value for any hypothesis *H* (hypnosis; belief in a hypothesis is constituted by the sum of the masses of all sets enclosed by subjective probabilities) by given evidence, as shown in

(3) $$\begin{array}{c} Bel\left(H\right)=\sum_{U\in H}m\left(U\right). \end{array}$$

The degree of trust depends on the reliability of the given evidence and on the overall environmental impact; the ratio of the degree is expressed by *e*.

(4) $$\begin{array}{c} m^{e}\left(A\right)=\left\{\begin{array}{ll} \left(1-r\right)m\left(A\right), & A\subset \theta ,\\ r+\left(1-r\right)m\left(\theta \right), & A=0, \end{array}\right. \end{array}$$

where *r* is a value between 0 and 1 and is true if *r* = 0 and false if *r* = 1. The DS calculates the value of a new belief through the process of fusion between different evidence. Thus, the convergence between the evidence can be expressed as (5); if *X*∩*Y* = ∅, then the convergence value of the two evidence is zero.

(5) $$\begin{array}{c} m_{1}⨁m_{2}\left(A\right)={\left(1-p\right)}^{-1}\sum_{X⋂Y=A}m_{1}\left(X\right)m_{2}\left(Y\right), \end{array}$$

(6) $$\begin{array}{c} p=\sum_{X\cap Y=\emptyset }m_{1}\left(X\right)m_{2}\left(Y\right). \end{array}$$

The DS expresses the confidence measure for *H* as [Bel (*H*), Pls (*H*)] and the term as the interval. This interval is called the “evidential interval.” Plausibility (Pls) means the extent to which the hypothesis is not negated based on evidence (empty period except for true and false intervals), which means the maximum likelihood of being trusted. Bel has a range from 0 to 1 (true and false), Pls can be defined as in (7) and has a value of [0,1]. Likewise, the likelihood values can express the process of fusion from multiple evidence as well as the fusion of belief values.

(7) $$\begin{array}{c} Pls\left(H\right)=1-Bel\left(\neg H\right), \end{array}$$

(8) $$\begin{array}{c} Pls\left(U\right)=\left(\left(\left({Pls}_{1}⨁{Pls}_{2}\right)⨁{Pls}_{3}\right)⨁\cdots \right)⨁{Pls}_{n}. \end{array}$$

In this study, three output data predicted from a multiclassifier were fused and calculated. The calculation process using DS shown in the figure as *DBN#1* (initial PSA) was set to *m*_1_, *DBN#2* (Gleason score) was set to *m*_2_, and *DBN#3* (clinical T stage) was set to *m*_3_. For the output data, the empty set of each of *m*_1_, *m*_2_, and *m*_3_ is given by

(9) $$\begin{array}{c} m_{1}\left(\emptyset \right)=1-\left(m_{1}\left(OCD\right)+m_{1}\left(NOCD\right)\right), \end{array}$$

(10) $$\begin{array}{c} m_{2}\left(\emptyset \right)=1-\left(m_{2}\left(OCD\right)+m_{2}\left(NOCD\right)\right), \end{array}$$

(11) $$\begin{array}{c} m_{3}\left(\emptyset \right)=1-\left(m_{3}\left(OCD\right)+m_{3}\left(NOCD\right)\right). \end{array}$$

As described above, *m*_1_, *m*_2_, and *m*_3_ were obtained, and then *m*_4_ is combined. The combination of *m*_4_ is shown in

(12) $$\begin{array}{c} m_{4}\left(OCD\right)=m_{1}\left(OCD\right)⨁m_{2}\left(OCD\right)⨁m_{3}\left(OCD\right)=\frac{\sum_{OCD\cap NOCD=OCD}m_{1}\left(OCD\right)m_{2}\left(OCD\right)m_{3}\left(OCD\right)}{\sum_{OCD\cap NOCD=\emptyset }m_{1}\left(OCD\right)m_{2}\left(OCD\right)m_{3}\left(NOCD\right)},\\ m_{4}\left(NOCD\right)=m_{1}\left(NOCD\right)⨁m_{2}\left(NOCD\right)⨁m_{3}\left(OCD\right)=\frac{\sum_{OCD\cap NOCD=NOCD}m_{1}\left(NOCD\right)m_{2}\left(NOCD\right)m_{3}\left(NOCD\right)}{\sum_{OCD\cap NOCD=\emptyset }m_{1}\left(NOCD\right)m_{2}\left(NOCD\right)m_{3}\left(NOCD\right)}. \end{array}$$

Next, the interval of the pass and fail of the evidential interval are summarized as

(13) $$\begin{array}{c} Bel\left(OCD\right)=m_{3}\left(OCD\right),\\ Pls\left(OCD\right)=1-\neg Bel\left(OCD\right),\\ Bel\left(NOCD\right)=m_{3}\left(NOCD\right),\\ Pls\left(NOCD\right)=1-\neg Bel\left(NOCD\right). \end{array}$$

As described above, the evidential interval section is constructed for OCD and NOCD, and the higher probability value of OCD and NOCD was set as the final output value.

Uncertainty data processing is a critical issue in the data fusion process. The DS and the Bayesian methods were compared to deal with this uncertainty. Unlike Bayesian inference, DS can contribute different levels of information to each source. In addition, a popular approach to data fusion has been established; unlike the Bayesian method, reliability can be assigned to all subsets of a hypothetical group, making it possible to form distributions for all subsets [31].

## 3. Result

### 3.1. Dataset Description

The characteristics of the initial PSA variable in the OCD and NOCD groups are shown in Table 1. Among the 6345 men, the average PSA levels in the OCD and NOCD groups in the training set were 9.535 and 18.606 ng/mL, respectively. In general, the level in the OCD group was higher, and the validation set also shows a difference of 9.377 and 17.899 ng/mL in the OCD and NOCD groups, respectively. The difference in values between the training and validation sets was not large. Although a high number of patients were observed at maximum, this is not a problem for analysis because they were only a fraction of the outlier compared to the mean.

The Gleason scores in the OCD and NOCD groups are shown in Table 2. Patients with OCD had a high Gleason score of 6. The NOCD group had scores of 6 or more. The difference between the OCD and NOCD groups was significant. In the scores below 5, OCD is more distributed than NOCD, and even more than 9 patients showed more NOCD patients.

The clinical T stages in the OCD and NOCD groups are shown in Table 3. Most patients were T2+. T1a occurred only in patients with OCD. In addition, many patients that are distributed in OCD until T1+ and patients with T3+ belong to NOCD. Although all variables are bounded by OCD and NOCD, there are many patients who belong to the same distributions.

### 3.2. DBN-DS Based Multiclassifier

**Figure 2:**
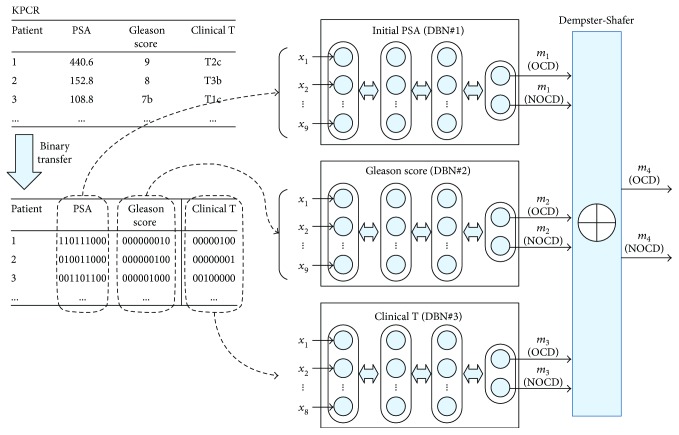
DBN-DS-based multiclassifier.

The proposed DBN and DS-based multiclassifier is shown in Figure 2. The training set was first changed to binary form. The initial PSA values were expressed as nine binary numbers based on the highest value (440 ng/mL). The Gleason score was composed of nine flags ranging from 3 to 10. The clinical T stage consisted of eight flags from T1a to T3b. The binary data of each of these variables was learned by the DBN classifier; that is, the first DBN consisted of nine input nodes because it was the input data of the initial PSA binary data. The output nodes of all classifiers were composed of two so that OCD and NOCD could be calculated with probability. The DBN consisted of three RBM layers, with the number of nodes of each RBM the same as the number of input nodes. Unsupervised learning was performed 100 times in total, while supervised learning using back propagation was performed 1000 times. Finally, we calculated the probability of the output variables as DS and determined the final number of *m*_4_(OCD) and *m*_4_(NOCD) as the final outputs.

### 3.3. Experiments

To evaluate the DBN-DS-based multiclassifier, the entire data set was divided into a 70% training set and a 30% testing set. The control groups included Decision Tree C4.5, naive Bayesian (NB), logistic regression (LR), back propagation (BP), support vector machine (SVM), random forest (RF), deep belief network, and Partin tables. The experiments compared the sensitivity, specificity, accuracy, and area under the curve (AUC) using confusion matrix [31] and receiver operating characteristics (ROC) curve analysis [32]. The experimental results of confusion matrix are shown in Table 4.

**Figure 3:**
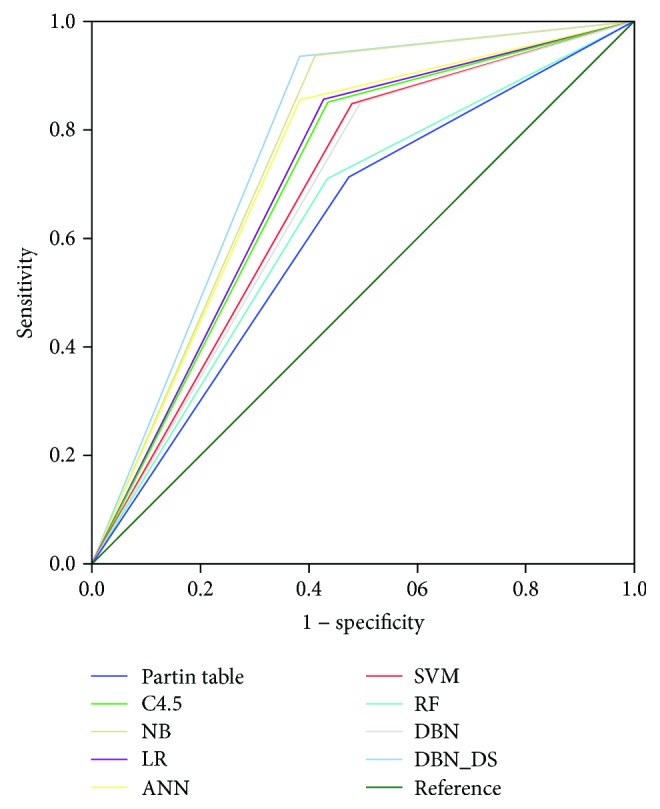
ROC curve results of all classification methods using the validation set.

In general, the results from a training set are better than those of a validation set because of differences in dataset volumes. Sensitivity was defined as the probability of correctly matching NOCD. Because NOCD has less data than OCD, it is difficult to match. The proposed method has a 61.77% improved performance compared to those of the other models. In other words, the probability of matching NOCD is very important because it is a prediction of the risk of the pathology stage. Specificity was defined as the probability of correctly matching OCD. NB had the highest specificity, with 93.78%, but its sensitivity was low. The proposed method showed 93.56% higher performance than those of the other models. The accuracy was defined as the probability of predicting both NOCD and OCD. The proposed model had the highest accuracy, at 81.27%. The AUCs are shown in Figure 3 and Table 5.

The ROC curve has the highest DBN-DS of 0.777. The error of all models was about 0.01, and the *p* values were all 0.000, so the experimental results of the ROC curves were usable. The DBN-DS predicted each of the three classifiers constructed for each variable separately and combined them into one. In this paper, we propose a new classification method for the classifier. The proposed method is based on the classification of two classifiers. In addition, as the DS computes probability, if one classifier predicts NOCD at a high number and the two classifiers predict a low number for OCD, then the NOCD is finally predicted based on the belief value of the DS algorithm.

**Figure 4:**
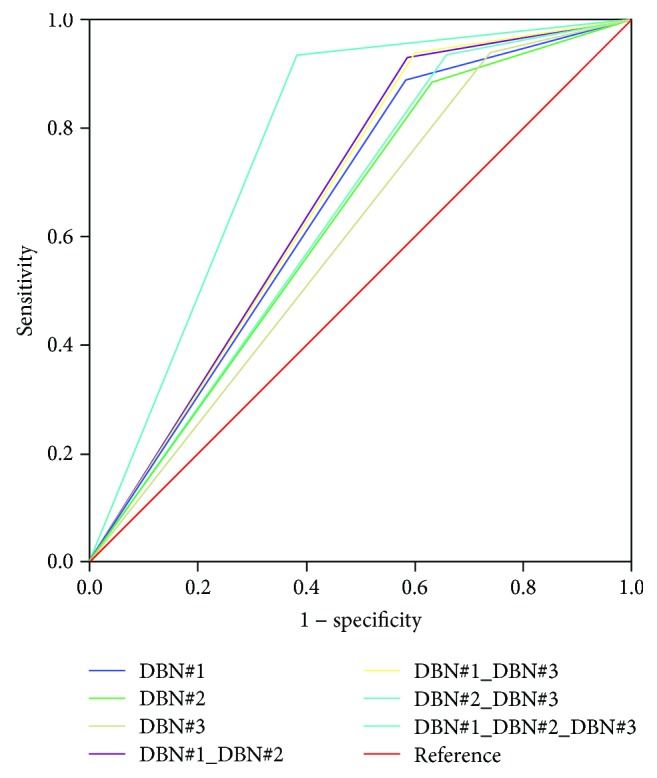
ROC curve results of DBN-DS using a validation set.

Next, the DBN-DS was evaluated. The result of the confusion matrix for DBN-DS is shown in Table 6. In addition, the results of the ROC curve analysis are shown in Figure 4 and Table 7. DBN#1 learned the initial PSA. DBN#2 learned the Gleason score, while DBN#3 learned the clinical T stage.

Among the three variables, the initial PSA level had the highest prediction rate. The PSA level is closely related to pathologic stage and is the most important parameter in prostate cancer. Variables combined with PSA showed a high prediction rate. In other words, the reason for the high prediction rate was that the Gleason score and clinical T stage also affect the pathology. However, the combination of Gleason score and clinical T stage had a lower accuracy than that predicted by the initial PSA level alone. The two variables are uncertain because they are diagnosed according to the doctor's experience. However, when combined with PSA level, the performance was much higher. In this study, we found that initial PSA was the most important predictor, and that the Gleason score and clinical T stage were also important predictors.

## 4. Discussion and Conclusion

Prediction models for pathology staging of prostate cancer are based on clinical tests and can be used to predict the spread of cancer. It is possible to diagnose cancer more precisely at the postoperative, pathological stage and to determine the degree of metastasis of prostate cancer.

We proposed a DBN-DS-based multiclassifier approach to predict the pathologic stage of prostate cancer. The proposed method provides a predictive model to improve accuracy through deep learning and information fusion based on the relationship between data measured using clinical tests. The inputs include initial PSA level, Gleason scores, and clinical T stage variables. The output can be OCD or NOCD in pathological staging (pT). This approach was evaluated using an existing validated patient dataset that included 6345 patient records from the KPCR database, which collected data from six tertiary medical institutions.

The performance of the proposed DBN-DS was compared with that of the NB, LR, BPN, SVM, RF, DBN, and Partin tables. The results showed that the proposed DBN-DS had better sensitivity and accuracy than all other methods.

In a recent pathological staging methodology study, Cosma et al. [4] use a neuro-fuzzy model, with an approach similar to ours. The results also indicated that the neural network-fuzzy-based computational intelligence learning approach is suitable for prostate cancer staging and exceeds the performance of the Partin tables. The neuro-fuzzy model and our proposed method aim to predict whether a patient has OCD (pT2) or NOCD (pT3+). All methods use the initial PSA level, Gleason scores, and clinical T stage to predict the pathologic stage of prostate cancer, but the DBN-DS predicts more patient data than other studies. In addition, it is possible to learn more deeply through the DBN-DS in order to improve the prediction performance in the existing DBN. The neuro-fuzzy model obtained an area under the curve (AUC) of 0.812, while the nomogram of the AJCC achieved an AUC of 0.582. Our proposed DBN-DS achieved an AUC of 0.777, compared to 0.620 for the Partin tables. This result is similar to that reported by Cosma et al. [4], although different data sets were used for each study; however, they show a high consistency with the results of the present study.

Currently, the proposed DBN-DS method is implemented as a research tool. Once the clinical evaluation is completed, the proposed tool will be developed as an easy-to-use clinical decision support system that can be accessed by clinicians.

## Figures and Tables

**Table 1:** Summary of initial PSA by pathology stage (organ-confined or non-organ-confined disease) in 6345 patients with clinically localized prostate carcinoma.

	Training set (*n* = 4039)		Validation set (*n* = 2306)	
	OCD (*n* = 2478)	NOCD (*n* = 1561)	OCD (*n* = 1414)	NOCD (*n* = 892)
Initial PSA				
Minimum	4	4	4	4
Maximum	160	440.60	81.13	164
Average	9.535 (0.173)	18.606 (0.622)	9.377 (0.197)	17.889 (0.653)

**Table 2:** Distribution of Gleason scores by pathology stage (organ-confined or non-organ-confined disease) in 6345 patients with clinically localized prostate carcinoma.

Gleason score	Training set (*n* = 4039)		Validation set (*n* = 2306)	
	OCD (%) (*n* = 2478)	NOCD (%) (*n* = 1561)	OCD (%) (*n* = 1414)	NOCD (%) (*n* = 892)
3	3 (0.12)	0 (0.00)	0 (0.00)	1 (0.11)
4	5 (0.20)	5 (0.33)	6 (0.42)	1 (0.11)
5	6 (0.24)	11 (0.73)	8 (0.57)	1 (0.11)
6	1342 (54.16)	378 (24.93)	785 (55.52)	235 (26.35)
7 (3 + 4)	565 (22.80)	386 (25.46)	306 (21.64)	218 (24.44)
7 (4 + 3)	266 (10.73)	277 (18.27)	160 (11.32)	159 (17.83)
8	238 (9.60)	326 (21.50)	119 (6.42)	174 (19.51)
9	46 (1.88)	147 (9.70)	28 (1.98)	95 (10.65)
10	7 (0.28)	31 (2.04)	2 (0.14)	8 (0.90)

**Table 3:** Distribution of clinical T stage by pathology stage (organ-confined disease and non-organ-confined disease) in 6345 patients with clinically localized prostate carcinoma.

Clinical T stage	Training set (*n* = 4039)		Validation set (*n* = 2306)	
	OCD (%) (*n* = 2478)	NOCD (%) (*n* = 1561)	OCD (%) (*n* = 1414)	NOCD (%) (*n* = 892)
T1a	9 (0.36)	0 (0.00)	3 (0.21)	0 (0.00)
T1b	107 (4.32)	49 (3.23)	74 (5.23)	18 (2.02)
T1c	988 (39.87)	410 (27.04)	556 (39.32)	225 (25.22)
T2a	691 (27.89)	380 (25.07)	417 (29.49)	241 (27.02)
T2b	278 (11.22)	161 (10.62)	151 (10.68)	97 (10.87)
T2c	234 (9.44)	224 (14.78)	126 (8.91)	127 (14.24)
T3a	150 (6.05)	233 (15.37)	66 (4.67)	135 (15.13)
T3b	21 (0.85)	104 (6.86)	21 (1.49)	49 (5.49)

**Table 4:** Experimental results of all classification methods between the training and validation sets.

	Training set			Validation set		
	Sensitivity	Specificity	Accuracy	Sensitivity	Specificity	Accuracy
Partin table	45.96%	88.44%	70.52%	52.69%	71.36%	64.14%
C4.5	64.46%	91.32%	80.46%	56.61%	85.22%	74.15%
NB	64.46%	93.30%	81.64%	58.86%	93.78%	80.27%
LR	60.65%	92.16%	79.42%	57.29%	85.64%	74.67%
BPN	63.90%	92.02%	80.60%	61.66%	85.57%	76.32%
SVM	52.13%	89.21%	74.35%	52.13%	84.87%	72.20%
RF	57.37%	86.43%	74.86%	56.73%	70.93%	65.44%
DBN	44.61	88.04	71.65%	50.56%	85.01%	71.68%
DBN-DS (proposed)	65.13%	94.29%	82.60%	61.77%	93.56%	81.27%

**Table 5:** Results of a DBN-DS confusion matrix comparing the training and validation sets.

	Variable	Training set			Validation set		
		Sensitivity	Specificity	Accuracy	Sensitivity	Specificity	Accuracy
DBN#1	Initial PSA	38.57%	91.39%	70.78%	41.93%	88.68%	70.60%
DBN#2	Gleason score	32.51%	89.18%	67.26%	37.00%	88.47%	68.56%
DBN#3	Clinical T stage	21.19%	94.20%	65.96%	26.23%	93.85%	67.69%
DBN#1, DBN#2	Initial PSA, Gleason score	41.48%	93.71%	73.50%	41.48%	93.00%	73.07%
DBN#1, DBN#3	Initial PSA, Clinical T stage	40.02%	94.55%	73.46%	40.02%	93.85%	73.03%
DBN#2, DBN#3	Gleason score, Clinical T stage	34.19%	94.91%	71.42%	34.19%	93.49%	70.56%
DBN#1, DBN#2, DBN#3 (proposed)	Initial PSA, Gleason score, Clinical T stage	65.13%	94.29%	82.60%	61.77%	93.56%	81.27%

**Table 6:** Detailed ROC curve analysis results of all classification methods using the validation set.

	AUC	*p* value	95% confidence interval	
			Lower bound	Upper bound
Partin table	0.620 ± 0.012	0.000	0.597	0.644
C4.5	0.709 ± 0.012	0.000	0.686	0.731
NB	0.763 ± 0.011	0.000	0.741	0.785
LR	0.715 ± 0.012	0.000	0.692	0.737
ANN	0.736 ± 0.012	0.000	0.714	0.758
SVM	0.685 ± 0.012	0.000	0.662	0.708
RF	0.638 ± 0.012	0.000	0.615	0.662
DBN	0.678 ± 0.012	0.000	0.655	0.701
DBN-DS	0.777 ± 0.011	0.000	0.755	0.798

**Table 7:** Detailed ROC curve result of DBN-DS using validation set.

	AUC	*p* value	95% confidence interval	
			Lower bound	Upper bound
DBN#1	0.653 ± 0.012	0.000	0.629	0.677
DBN#2	0.627 ± 0.012	0.000	0.603	0.651
DBN#3	0.600 ± 0.012	0.000	0.576	0.625
DBN#1, DBN#2	0.672 ± 0.012	0.000	0.649	0.696
DBN#1, DBN#3	0.669 ± 0.012	0.000	0.646	0.693
DBN#2, DBN#3	0.638 ± 0.012	0.000	0.614	0.663
DBN#1, DBN#2, DBN#3 (proposed)	0.777 ± 0.011	0.000	0.755	0.798
